# Controlled tip wear on high roughness surfaces yields gradual broadening and rounding of cantilever tips

**DOI:** 10.1038/srep36972

**Published:** 2016-11-11

**Authors:** Daan Vorselen, Ernst S. Kooreman, Gijs J. L. Wuite, Wouter H. Roos

**Affiliations:** 1 Department of Physics and Astronomy and LaserLab, Vrije Universiteit, Amsterdam, 1081 HV, The Netherlands; 2 Department of Oral Function and Restorative Dentistry, Academic Centre for Dentistry Amsterdam (ACTA), Research Institute MOVE, University of Amsterdam and Vrije Universiteit, Amsterdam, 1081LA, The Netherlands; 3 Moleculaire Biofysica, Zernike Instituut, Rijksuniversiteit Groningen, Nijenborgh 4, 9747 AG Groningen, The Netherlands

## Abstract

Tip size in atomic force microscopy (AFM) has a major impact on the resolution of images and on the results of nanoindentation experiments. Tip wear is therefore a key limitation in the application of AFM. Here we show, however, how wear can be turned into an advantage as it allows for directed tip shaping. We studied tip wear on high roughness polycrystalline titanium and diamond surfaces and show that tip wear on these surfaces leads to an increased tip size with a rounded shape of the apex. Next, we fitted single peaks from AFM images in order to track the changes in tip radius over time. This method is in excellent agreement with the conventional blind tip reconstruction method with the additional advantage that we could use it to demonstrate that the increase in tip size is gradual. Moreover, with our approach we can shape and control the tip size, while retaining identical chemical and cantilever properties. This significantly expands the reproducibility of AFM force spectroscopy data and is therefore expected to find a wide applicability.

Atomic force microscopy (AFM) is a widely used tool for nanoscale imaging and force spectroscopy in material science, biology^1^ and lithography^2^,^3^. Using AFM in imaging mode, the size and geometry of the tips are critical for resolution, since the recorded image is always a convolution of the sample and the tip. Furthermore, in AFM force spectroscopy studies such as nanoindentation, the tip size and shape are critical for the observed response of the investigated material^4^. Therefore, knowledge and control of tip size is not only essential for image analysis, but also for the calculation of material properties, such as elastic moduli^4^,^5^. During AFM experiments, tips are continuously exposed to forces. Especially in lithography and nanoscale manufacturing applications this can change the tip size, i.e. tip wear, and thus influencing the results of these experiments. Therefore, AFM tip wear is a process that is currently subject of extensive research, using various tip materials and AFM operating modes^6^,^7^. Tip wear can occur by several mechanisms, such as adhesive wear^8^, abrasive wear^8^, fracture^9^,^10^ and plastic deformation^11^. Recently, it was found that nanoscale wear with low forces can be described accurately based on an atom-by-atom attrition process, which implies a very gradual wear mechanism^12^,^13^,^14^,^15^.

For characterization of the tip size and geometry in wear studies often scanning electron microscopy (SEM) is used^6^,^7^. However, it is time consuming to image tips at regular intervals during the wear process using SEM. Furthermore, some tip materials that are often used for AFM (*e.g*. silicon nitride) show little electrical conductivity and can therefore only be tracked at low resolution using SEM. An alternative is to use AFM itself for determining the tip size. Measuring adhesion forces^12^,^15^, reverse imaging^8^, blind tip reconstruction^16^ or spectral analysis of surface roughness^17^ can be used for finding the tip radius. An advantage of these methods is that tip size information can be obtained *in situ*. Additionally, blind tip reconstruction and reverse imaging provide 3D information of the geometry. Recently, different characterization methods including AFM blind tip reconstruction and SEM were compared and found to agree well^10^.

Finally, for many applications of AFM the role of tip size is not clear. To investigate the role of tip size, it is essential to have identical cantilevers, chemical properties and general shape of the tip, but different tip sizes. Since tip wear under low forces is found to be caused by a gradual atom-by-atom process^12^,^13^,^14^,^15^, tip wear could potentially also be applied for tip shaping.

Here, we study AFM tip wear of silicon nitride tips on high roughness titanium (Ti) and ultra nanocrystalline diamond (UNCD) surfaces in order to understand the wear process and to demonstrate the use of tip wear as a method for shaping of the tip. High roughness surfaces represent an important class of samples for AFM studies, *e.g*. superhydrophobic materials^18^ and polycrystalline surfaces^19^,^20^. The high roughness of the sample makes it ideal to use it for blind tip reconstruction^16^. The titanium sample has very sharp features and is commonly used as a sample for tip reconstruction. The hardness of UNCD makes it an ideal material for tip wear studies, since it wears the tip without wearing itself. We show that wear on these surfaces results in an increased tip size, while maintaining the rounded shape of the tip. Furthermore, we introduce a novel method for analyzing images to track AFM tip size. This method agrees well with blind tip reconstruction, while allowing more frequent tip size determination, showing that wear occurs gradually in our experiments. We thus show that tip wear on high roughness surfaces is not only of interest for a fundamental understanding of tip wear, but that it can also be applied as protocol for creating rounded tips of various sizes for applications in material science and biomechanics.

## Results

### Tip wear on high roughness surfaces

To study tip wear of silicon nitride tips on high roughness surfaces in contact mode both a flaked titanium surface and an UNCD surface were used. The roughness *R*_*rms*_ of these surfaces was determined from 2.5 × 2.5 μm images, taken at ~5 nN normal force. The surface roughness is *R*_*rms*_ = 39.4 ± 0.1 nm (s.e.m., N = 11) for the Ti surface and *R*_*rms*_ = 23.8 ± 0.6 nm (s.e.m., N = 5) for the UNCD surface.

**Figure 1:**
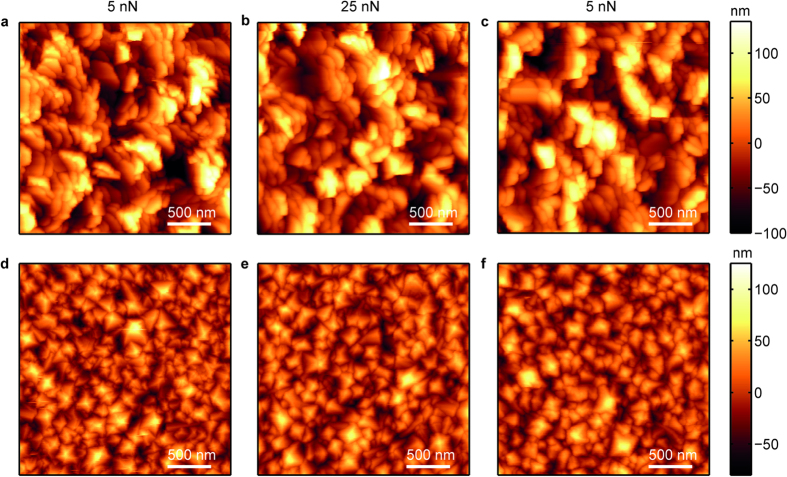
AFM images of polycrystalline surfaces. 2.5 × 2.5 μm images made at 1,024 × 1,024 pixel resolution. (**a–c**) Titanium surface. **(d–f**) UNCD surface. (**a**,**d**) Images made at 5 nN normal imaging force. (**b**,**e**) Images made at 25 nN normal imaging force. (**c**,**f**) Images made at 5 nN imaging force after wear experiments. Images (**c**,**f**) are made with the same tip as in (**b**,**e**) respectively. (**d**) corresponds to the same tip as (**e**,**f**) whereas a is a different tip than (**b**,**c**). Notice the decrease in resolution when comparing panels (**a**,**d**) with (**c**,**f**). The decrease in resolution is also visible from top to bottom in panel (**b**,**e**). Upper colorbar marks the height scale of (**a–c**). Lower color bar marks the height scale of (**d–f**).

**Figure 2:**
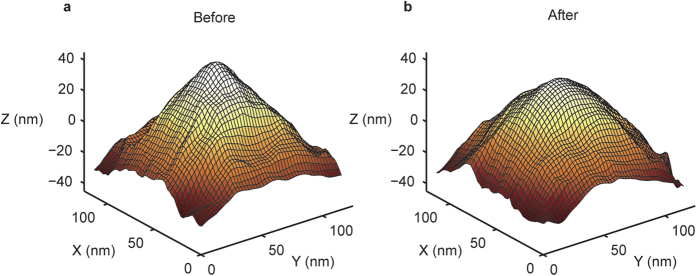
AFM tip images of a single tip created using blind tip reconstructions. (**a**) Reconstruction before and (**b**) after a wear experiment on a UNCD surface.

For determination of the tip geometry, blind tip reconstruction was used. The high roughness surfaces used for this study are ideal for this purpose, giving a large part of the tip that can be reconstructed. First an image was taken at ~5 nN normal force (Fig. 1a), for an estimate of the initial tip shape. Next, to increase the contact stress and hence the wear rate, the surfaces were imaged at ~25 nN (Fig. 1b,e). Finally, another image was taken at ~5 nN normal force for estimation of the tip shape of the worn tip (Fig. 1c,f). To exclude wear of the surface influencing blind tip reconstruction, different areas were imaged. The resolution of images made after tip wear (Fig. 1c,f) is visibly lower than the resolution before the wear experiment (Fig. 1a,d) indicating an increase of tip size. For blind tip reconstruction, images were flattened line by line and low pass filtered. Resultant tip shapes are shown in Fig. 2 and Supplementary Fig. S1. A reported disadvantage of blind tip reconstruction is that typically only a small region of the tip around the apex can be resolved (~10 nm)^10^,^11^, however by using the high roughness surfaces reported in this study we are able to reconstruct tip shapes up to 100 nm below the apex. Clearly, we can observe an increase in the radius of the tip, before and after wear. Furthermore, both before and after wear the tip geometry is rounded.

### Tip wear on high roughness surfaces leads to larger tips with similar shape

**Figure 3:**
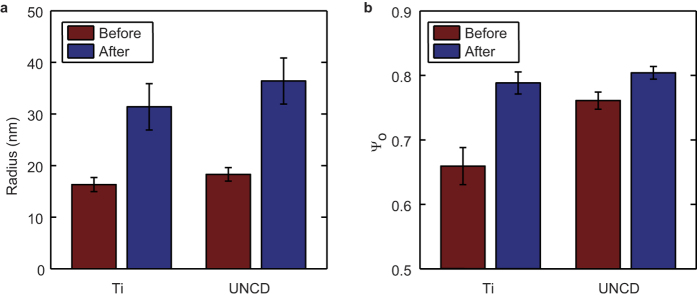
Radius and sphericity *Ψ*_*o*_ of new and worn silicon nitride tips. **(a**) Radius of curvature obtained from reconstructed tip images using blind tip estimation for both titanium (Ti) and UNCD surfaces before and after the wear experiment. (**b**) Quantification of the sphericity of the tips. An adjusted sphericity measure (open sphericity) was used which takes into account the open shape (see main text). Errorbars indicate standard error of the mean with N = 12 for the tips worn on the Titanium surface and N = 5 for the tips worn on the UNCD surface. The sphericity increased significantly for tips worn on both Ti and UNCD surfaces.

To quantify the increase in tip size we determined the radius of curvature of the top 15 nm of each tip. The mean radius obtained on the UNCD- and Ti surface is very comparable: 18 ± 1 nm (s.e.m., N = 5) and 16 ± 1 nm respectively (s.e.m., N = 12) (Fig. 3a). After the wear experiment we found a significant increase in the tip size on both surfaces: 36 ± 4 nm (s.e.m., N = 5) and 31 ± 4 nm (s.e.m., N = 6). To estimate the worn volume we created images before and after wearing the same tip on the UNCD surface. We created line profiles along the fast and slow scanning axes and overlaid them by maximizing the occupation of the area of the unworn tip by the worn tip, while the width of the worn tip was not allowed to exceed the unworn tip for any height (Supplementary Fig. S2). Subsequently, we measured the tip volumes for new and worn tips until the point the tip shapes have equal width using the disc method^10^,^13^. This allowed us to estimate the worn volumes (4.5 ± 1.3 × 10^4^ nm^3^), which corresponds to a wear rate of 88 ± 26 nm^3^ per μm slided distance. This wear rate is comparable to recent studies in which the wear mechanism was suggested to be atom-by-atom attrition^10^,^13^.

The increase in radius and rounded appearance of the worn tips, suggest that tip wear can be applied to create tips with increased radii, but similar shape and cantilever properties. Studies of tip wear using low roughness surfaces have mostly led to tips with a flattened apex^6^,^7^. It is likely that the high surface roughness used in the current study ensures that the surface is in touch with – and hence wears – the tip over a larger surface area, *i.e*. not only at the apex. To quantify the roundness of our tips, we measured the sphericity (*Ψ* = *π*^*1*/*3*^(*6* *V*)^*2*/*3*^*/A*), where *V* is the measured volume and A is the measured surface area of the tip. The sphericity is a measure defined as the surface area of a sphere of the same volume as the measured particle divided by the measured surface area of the particle and hence is 1 for a sphere. However, for an open shape the sphericity can exceed 1, therefore we introduce an adjusted measure: the open sphericity *Ψ*_*o*_ = (*18π*)^*1*/*3*^ *V*^*2*/*3*^*/A*. *Ψ*_*o*_ equals its maximum 1 for an open hemisphere. For a paraboloid, a frequently used shape to describe the geometry of AFM tips, *Ψ*_*o*_ ≈ 0.93 when measured until the *R*_*t*_ below the apex, where *R*_*t*_ is the radius of curvature at the apex. We measured the open sphericity from the tip apex to *R*_*t*_ below the apex from reconstructed tip images. For new tips, before our wear experiment, we found that *Ψ*_*o*_ is 0.76 ± 0.01 (s.e.m., N = 5) for tips reconstructed from images of the UNCD surface and 0.66 ± 0.03 (s.e.m., N = 12) for tips reconstructed from the Ti surface (Fig. 3b). The Ti surface has sharper features and therefore leads to higher resolution of the tip, whereas the more rounded UNCD surface leads to higher sphericity estimates (Supplementary Note). For worn tips we found 0.79 ± 0.01 (s.e.m., N = 5) and 0.80 ± 0.01 (s.e.m., N = 5) for Ti and UNCD surfaces respectively, marking a significant increase of the sphericity of the tip after our wear protocol on both surfaces. In addition, the rounded shape implies that wear occurs through a gradual process affecting a relatively large area of the tip.

### Fast *in situ* tip wear tracking reveals the gradual nature of wear

**Figure 4:**
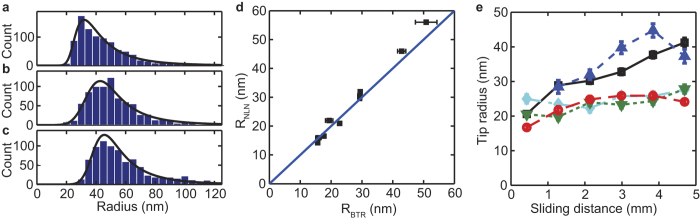
Tip radius determination from line profiles allows tip wear tracking over time. (**a–c**) Histograms showing typical distribution of peak radii from images. Data from UNCD surfaces. In black: normal-lognormal fits, for which the log-normal distribution parameters (attributed to the surface) were fit globally and are the same for all 3 histograms. Histograms were made using 1/6 image, which corresponds to a scanning area of 0.4 × 2.5 μm. (**d**) Comparison of radii obtained using blind tip reconstruction and subsequent fitting of the obtained tip image (R_BTR_) and parabolic fitting of individual peaks in line profiles along the fast scanning axes and subsequent fitting using a normal-lognormal distribution (R_NLN_). Both from UNCD surfaces (N = 10). Errorbars in x-direction represent the standard deviation of the tip radius measurements from reconstructed images in both scan directions (forward and reverse scan direction). Errorbars in y-direction represent 95% fitting parameter confidence intervals, assuming accurate determination of surface properties. Most errorbars are smaller than the marker size. The blue line represents y = x. (**e**) Tip radius versus sliding distance when scanning on a UNCD surface with 25 nN normal imaging force. 5 individual tips are visualized using various line styles and colors. Errorbars correspond to 95% confidence interval of the fitted mean, under the assumption that the surface properties were determined accurately. Average tip radius of the 5 tips increased from 22 ± 2 nm (s.e.m.) to 32 ± 3 nm (s.e.m.). (**a–c**) correspond to the first, second and fourth data point in black in panel (e) respectively.

To follow the evolution of tip size over time during wear experiments, we developed a method based on analysis of individual peaks in line profiles along the fast scanning axis of AFM images. Similar to frequently used SEM for studying tip shape, this method uses only 2D information on the tip shape. Individual peaks were fit using a parabolic function to obtain a radius of curvature. This method yielded on average ~5000 peaks for a 2.5 × 2.5 μm image. The peaks sizes that are found in this way are a combination of the width of sample peaks and the tip size. Therefore, we fitted distributions of peak sizes with the convolution of a normal distribution, attributed to the tip geometry, and a lognormal distribution, attributed to the sample (Fig. 4a–c). A lognormal distribution was previously shown to accurately describe grain size distribution in crystalline materials^21^. The surface properties (Supplementary Table S1) were obtained from a global fit, using only images obtained at low force setpoint (5 nN) with new tips. For subsequent fits these properties were kept constant. We compared the average tip radii with the estimates obtained from blind tip reconstruction and found excellent agreement for the tip radii measured on UNCD (correlation coefficient R > 0.995) (Fig. 4b). For tips measured on the Ti surface, the agreement was also good (R ~ 0.895) (Supplementary Fig. S3), illustrating the general applicability of this method.

The advantage of our method is that many peaks can be used to determine the radius and thus tip estimates can be made more frequently. This advantage in turn allowed us to track the change in tip size during the wear process. For the tracking the images created during wear experiments were split in 6 sections of equal length, and the tip radius was estimated for each (Fig. 4a–c). The distribution of obtained radii clearly shifted to higher values. The experiments on UNCD surfaces all showed an increase during 5 mm of sliding, but with various magnitudes (Fig. 4e). The same analysis was repeated for the tips on the Ti surface (Supplementary Fig. S3). The increase in radius of the tips showed a higher spread, but occurred gradual too. It was previously suggested that high surface roughness leads to abrasive tip wear^22^,^23^. However, in our case the increase in radius we observe appears to be gradual, which is not expected for abrasive wear.

## Discussion

In this study we investigated tip wear on high roughness surfaces. As previously mentioned, rough samples can have multiple advantages for studying tip wear, such as tracking of the imaging performance of the tip and the possibility to use the sample for blind tip estimation^10^. An additional benefit of high roughness samples is that the tip surface contact area is dominated by the surface and is likely to change little during the wear experiment. Indeed, we do not observe a strong decrease in wear rate. With low roughness samples the size of the contact area is dominated by the tip and therefore leads to decreased contact stresses and wear rate over time^12^. Increase of contact area is predicted to complicate the observed behavior by leading to different regimes during wear^12^, a complication which seems absent in our approach.

Since tip size can strongly affect AFM measurements, there has been a strong recent interest in developing methods for *in situ* tip size determination. These tools are based on *in situ* EM^13^, surface topography spectral analysis^17^, cantilever dynamics^24^,^25^ or the capacitor function of the tip-sample system^25^. With this study we have added a method to the currently available toolbox, in particular we added a method for fast *in situ* tip size determination based on statistical analysis of peaks in AFM images. Like some other recent methods^17^,^24^, the currently presented method does not require complex instrumentation and is not limited to conductive AFM tips. Our method is especially suited for hard high roughness surfaces, where no change of samples is required. Also, for biological applications our approach will be of interest, since, with the exception of for instance ref. 17, generally other approaches have not been shown to work for soft cantilevers (<0.1 N/m), which are often used for biological studies.

Tip shaping has previously been proposed as a tool to increase the effective radius of the AFM tip on mica^24^. However, for some applications of AFM, the tip shape, in addition to its size, is critical for the observed behavior. A clear example of such an application is AFM nanoindentation. Nanoindentation has become a widespread technique to study the mechanical properties of materials^26^ including biomaterials, such as viruses^27^ and cancer cells^28^. The observed mechanical behavior strongly depends on the increase in contact area of the tip and the sample, and thus the tip shape is critical for the observed response^4^. We expect that our method, which ensures a spherical tip apex, will be of use for determining the effect of tip size in nanoindentation studies and furthermore will be instrumental in obtaining more reproducible results, by ensuring that similar tip geometries are used to compare samples.

For the purpose of tip shaping it is important that the surface is much harder than the tip material, making high roughness UNCD, with a hardness of ~88 GPa^29^, a good choice. Our high roughness Ti surface has a hardness (7–18 GPa^30^,^31^,^32^, assuming an oxidized layer) comparable to or lower than the silicon nitride tips (~18 GPa)^33^. Therefore, the increase in radius of these tips is possibly due to addition of Ti material to the tip as well as of loss of tip material, possibly changing the chemical properties of the tip. This means that use of high roughness UNCD is preferred for tip shaping.

In summary, tip wear on high roughness surfaces can be applied to create tips of different radii, while maintaining spherical geometry. Moreover, we introduce a method of estimating the radius from individual peaks in line profiles, and show that it is very consistent with the radius obtained by blind tip estimation. This method makes use of more surface features and therefore allows the tracking of tip size over smaller areas and thus more frequently than blind tip estimation. Using this method, we demonstrated that tip wear in our study is gradual, which is consistent with other recent studies using low contact force^10^,^12^,^13^,^15^. To conclude, we expect that our approach will be useful for discovering the impact of tip size in many applications of atomic force microscopy imaging and force spectroscopy.

## Methods

### AFM wear experiments

Experiments were performed on a Bruker bioscope catalyst AFM setup. Olympus 0.1 Nm^−1^ cantilevers (OMCL-RC800PSA) with silicon nitride tips were used. The tips have a nominal tip radius of 15 nm, according to the manufacturer. Stiffness of individual cantilevers was calibrated using thermal tuning. Measurements were performed in contact mode on UNCD Aqua 100 surfaces (Advanced Diamond Technologies, Inc.) or a titanium roughness sample (Bruker). Root mean squared roughness (*R*_*RMS*_) of these surfaces was calculated as 
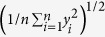
, where *y*_*i*_ is the height at pixel *i*. All recorded images were 2.5 × 2.5 μm in size at a resolution of 1024 × 1024 pixels, for a total sliding distance of ~5.1 mm. Scan rate was 0.2 Hz, resulting in a sliding velocity of 0.5 μms^−1^. Normal imaging force during wear experiments was 25 nN, for images for blind tip reconstruction 5 nN.

### Blind tip estimation

Blind tip estimation was performed with software from the AFM manufacturer (NanoScope Analysis). Images were flattened, low pass filtered and the tip estimation was performed using spike rejection (sigma mult 7) and discontinuity rejection (sigma mult 3). End radius (*R*_*t*_) was then estimated by fitting a circle to the circumference of the tip 15 nm below the tip apex and subsequently fitting a sphere through this circle and the tip apex. For sphericity calculation home-written MATLAB software was used. The region from *R*_*t*_ below the apex of the tip till the apex was used. Surface area was calculated by triangulation of the surface. Volume was calculated using numerical integration by the trapezoidal method using built-in MATLAB functionality.

### 2D tip wear tracking

For 2D tip wear tracking line profiles were taken along fast scanning axes of processed (flattened and low pass filtered) images. Subsequently, line profiles were analyzed using home-built MATLAB software. Peaks were detected and selected based on a minimal height difference of 20 nm with neighboring minima. Individual peaks were then fitted from 15 nm below the apex using a parabolic function (y = ax^2^ + bx + c). The radius of curvature was determined as the radius of curvature in the peak of the parabola (1/2a). Obtained radii were fit using a convolution of a normal and a lognormal (NLN) distribution. The convolution of these distributions was calculated numerically and linear interpolation was used to find the probability of individual observations. For fitting a maximum-likelihood approach was used, in which first 5 UNCD images and 10 Ti images – all made with a new tip and at 5 nN force – were used to globally fit the surface parameters. Subsequent fits to obtain tip radius were performed with fixed surface properties.

## Additional Information

**How to cite this article**: Vorselen, D. *et al*. Controlled tip wear on high roughness surfaces yields gradual broadening and rounding of cantilever tips. *Sci. Rep*. **6**, 36972; doi: 10.1038/srep36972 (2016).

**Publisher’s note:** Springer Nature remains neutral with regard to jurisdictional claims in published maps and institutional affiliations.

## Supplementary Material

Supplementary Information
