# Supplementary material for: Controlled tip wear on high roughness surfaces yields gradual broadening and rounding of cantilever tips
Source: Sci Rep. 2016 Nov 11;6:36972. doi: 10.1038/srep36972 (PMC5105056; doi:10.1038/srep36972)
Supplement: Supplementary Information [file srep36972-s1.doc]

Supplementary Information

Controlled tip wear on high roughness surfaces yields gradual broadening and rounding of cantilever tips

*Daan Vorselen^1,2,†^, Ernst S. Kooreman^1,†^, Gijs J.L. Wuite^1,*,§^ and Wouter H. Roos^1,3,*,§^*

^1^Department of Physics and Astronomy and LaserLab, Vrije Universiteit, Amsterdam, 1081 HV, The Netherlands

^2^Department of Oral Function and Restorative Dentistry, Academic Centre for Dentistry Amsterdam (ACTA), Research Institute MOVE, University of Amsterdam and Vrije Universiteit, Amsterdam, 1081LA, The Netherlands.

^3^Moleculaire Biofysica, Zernike Instituut, Rijksuniversiteit Groningen, Nijenborgh 4, 9747 AG Groningen, the Netherlands

*[g.j.l.wuite@vu.nl](mailto:g.j.l.wuite@vu.nl) and [w.h.roos@rug.nl](mailto:w.h.roos@rug.nl)

^†^These authors contributed equally. ^§^These authors contributed equally.

**Supplementary Note**

We note that the apparent sphericity of a particle is expected to increase when the relative resolution (pixels/tip radius) increases. However, we simulated this effect by calculating the sphericity of hemispheres using various grid sizes and found an increase of ~1% for a change comparable to our measured tip size change, which is not big enough to explain our observed increase.


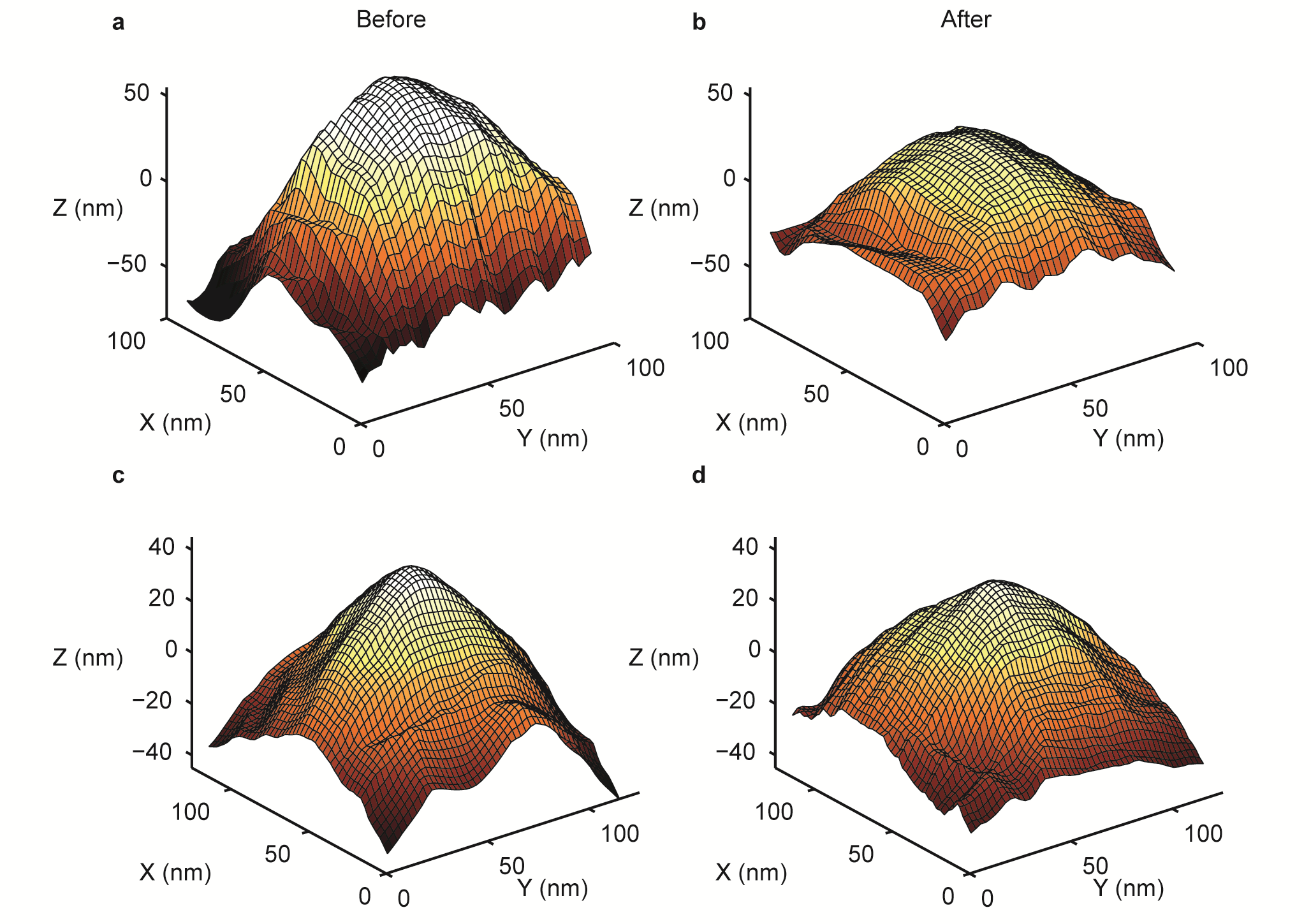


**Supplementary Figure S1. Additional AFM tip images created using blind tip reconstructions before and after wear experiments. (a,b**) Tip reconstructions from Ti surface images. (**a**) and (**b**) do not correspond to the same tip. (**c,d**) Tip reconstructions from UNCD surface images. (**c**) and (**d**) correspond to the same tip.


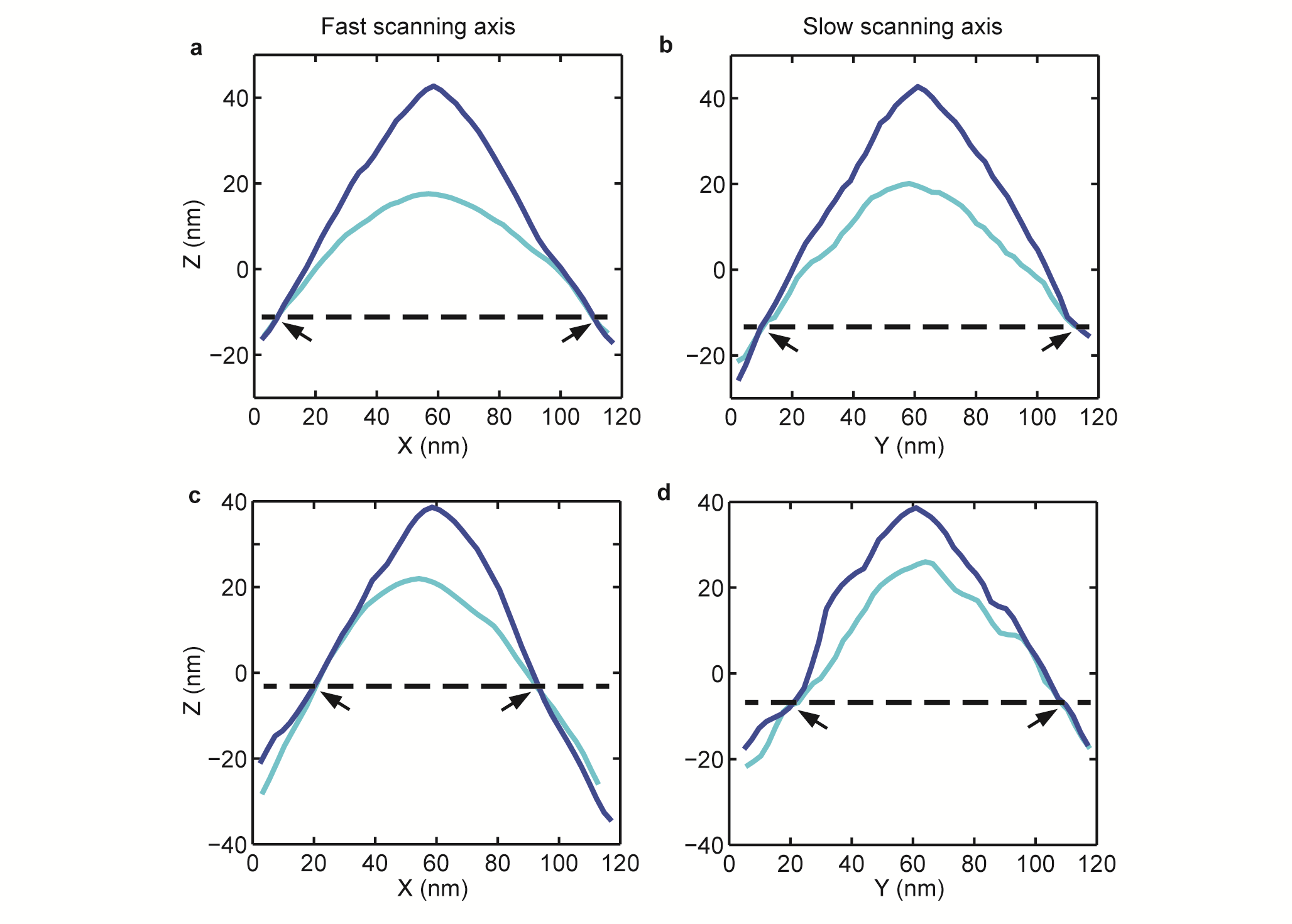


**Supplementary Figure S2. Determination of lost volume through tip wear on UNCD.** (**a,b**) Tip profile through the maximum of a tip along (**a)** the fast respectively (**b**) slow scanning axes of tip images created by blind tip reconstruction (BTR). In blue new tips and in cyan the same tip after wear experiments. Arrows and dashed line mark the height, where the width of the profiles is equal. The profile above this height was used for volume calculation. (**c,d**) Similar to (**a,b**), but for a different tip. Worn volume was obtained as follows: First the width of the line profile as function of the distance from the apex was determined. Linear interpolation was used to find points at equal height. By moving the line profiles along the Z-axis, the translation was determined that minimizes the smallest of the differences in width. Essentially, this results in maximizing the occupied surface area of the new tip by the worn tip, while the width of the worn tip is not allowed to exceed the width of the new tip for a given height. Then the volume was calculated above the point of equal width using the method of discs; the volume of cylinders with diameters equal to the width of the line profile and height equal to the spacing in Z between the data points were added. Finally, volume differences of the new and worn tip were calculated along both slow and fast scanning axis and averaged.


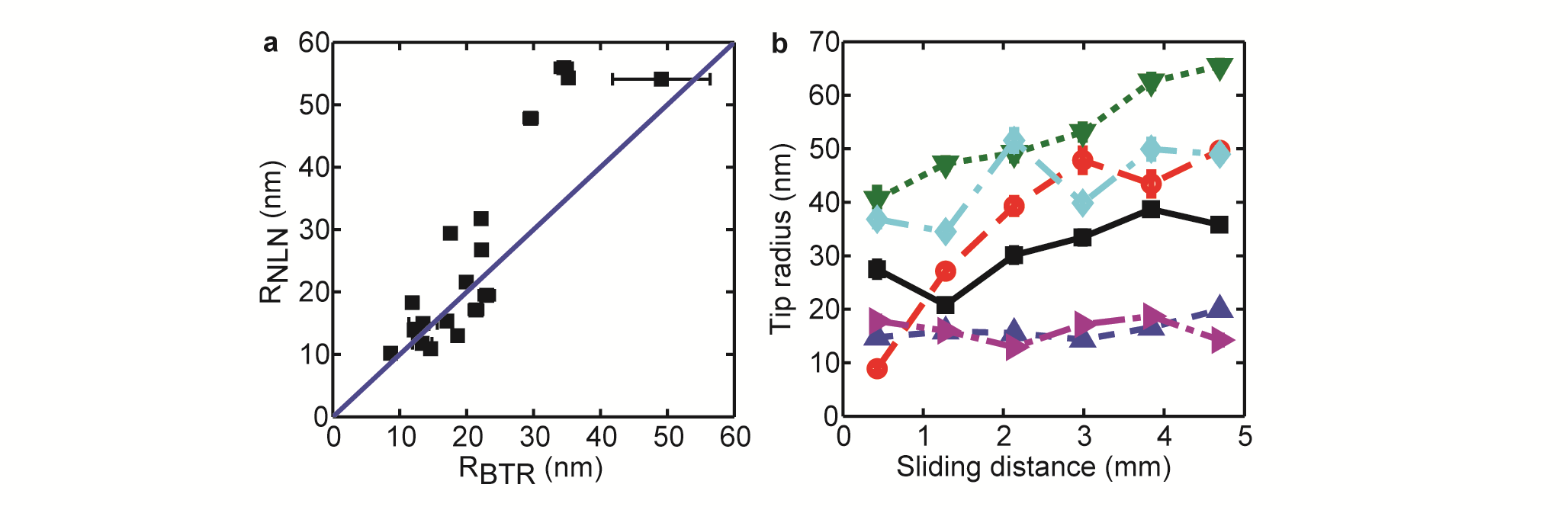


**Supplementary Figure S3: Tip radius determination from line profiles for tips worn on Ti surface.** Equivalent to Fig. 4d,e in the main text, but from tips imaged and worn on Ti surfaces. (**a**) Comparison of radii obtained using blind tip reconstruction and subsequent fitting of the obtained tip image (R_BTR_) and parabolic fitting of individual peaks in line profiles along the fast scanning axes and subsequent fitting using a normal-lognormal distribution (R_NLN_) (N = 18). Errorbars in x-direction represent the standard deviation of the tip radius measurements from reconstructed images in both directions (forward and reverse scan direction). Errorbars in y-direction represent 95% fitting parameter confidence intervals, assuming accurate determination of surface properties. Most errorbars are smaller than the marker size. The blue line represents y = x. (**b**) Tip radius versus sliding distance when scanning on a Ti surface with 25 nN normal imaging force. 5 individual tips are visualized using various line styles and colors. Errorbars correspond to 95% confidence interval of the fitted mean, under the assumption that the surface properties were determined accurately. Average tip radius of the 6 tips increased from 24 ± 4 nm (s.e.m.) to 39 ± 8 nm (s.e.m.).

| **Supplementary Table S1.** Estimated surface peak properties obtained with global fits. Parameters of the lognormal component of a fitted normal-lognormal distribution to observed radii of peaks in line profiles and derived mean and mode. Subscript g indicates properties derived using the global fit. Subscript i indicates individual fits of the same surfaces, with errors giving the standard error of the mean. Big differences can be due to local surface variations or imaging artifacts. | | | | | | | | | |
| --- | --- | --- | --- | --- | --- | --- | --- | --- | --- |
|  | N | μ_g_  _(nm)_ | μ_i_  _(nm)_ | σ_g_  _(nm)_ | σ_i_  _(nm)_ | Mean_g_  _(nm)_ | Mean_i_  _(nm)_ | Mode_g_  _(nm)_ | Mode_i_  _(nm)_ |
| **UNCD** | 5 | 2.93 | 2.99± 0.09 | 0.78 | 0.75± 0.08 | 26 | 27 ±2 | 10 | 12 ±2 |
| **Ti** | 10 | 2.83 | 3.09± 0.12 | 1.24 | 1.07± 0.06 | 37 | 43 ±6 | 3.6 | 8.1 ±2 |
